# Supplementary material for: Origin and Dynamics of Mycobacterium tuberculosis Subpopulations That Predictably Generate Drug Tolerance and Resistance
Source: mBio. 2022 Nov 8;13(6):e02795-22. doi: 10.1128/mbio.02795-22 (PMC9765434; doi:10.1128/mbio.02795-22)
Supplement: FIG S1 [file mbio.02795-22-s0001.pdf]

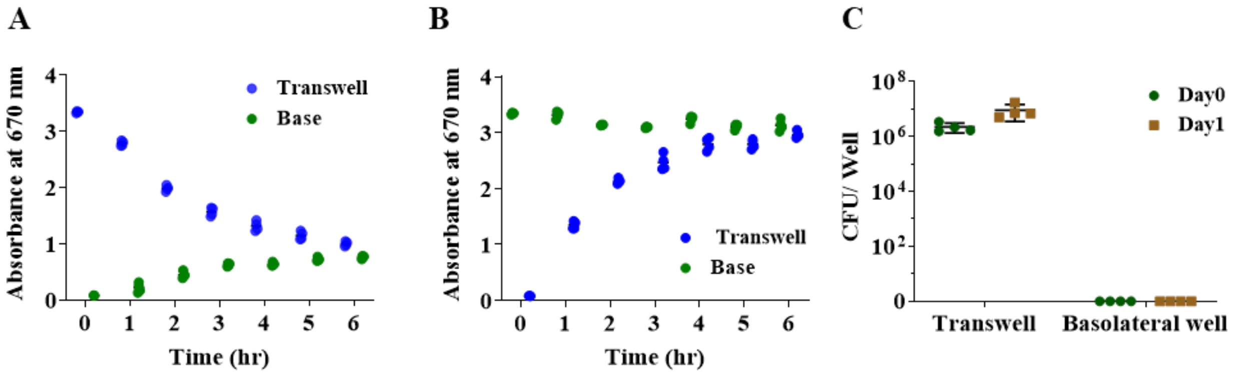

Fig. S1. Measuring diffusion across the 0.4 μm membrane separating each transwell from its paired basolateral well. Methylene blue was placed in each upper transwell A, or in each basolateral well B, and the concentration of dye in both wells was assessed by measuring the absorption at 670nm from each well over time. C, Approximately 2 x 10^6 *M. tuberculosis* CFU were placed in each upper transwell and the entire contents of each transwell and its paired basolateral well were cultured either immediately or after 24-hour incubation at 37 °C under shaking conditions. Each time point was tested in triplicate A, B or quadruplicate C. Means are plotted with standard deviation.
