# Supplementary material for: Origin and Dynamics of Mycobacterium tuberculosis Subpopulations That Predictably Generate Drug Tolerance and Resistance
Source: mBio. 2022 Nov 8;13(6):e02795-22. doi: 10.1128/mbio.02795-22 (PMC9765434; doi:10.1128/mbio.02795-22)
Supplement: FIG S2 [file mbio.02795-22-s0002.pdf]

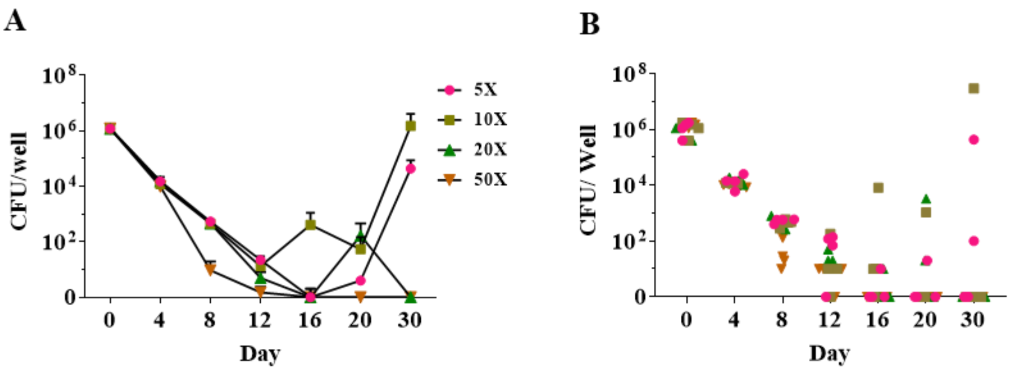

Fig. S2. Experiment 1 results - Steady state rifampicin exposure sterilizes *M. tuberculosis* cultures at high concentrations. Time-kill kinetics of actively growing *M. tuberculosis* cultures when subjected to steady-state rifampicin exposure at four different concentrations of rifampicin (see key showing rifampicin drug concentrations expressed as times (X) the minimal inhibitory concentration (MIC), where 1X the MIC = 0.01 µg/ml of rifampicin). A, shows the mean CFU from replicate cultures (Y axis) plated on drug free agar at different time points (X axis); B, shows the individual CFU from each replicate culture well of the same experiment.
