# Supplementary material for: Origin and Dynamics of Mycobacterium tuberculosis Subpopulations That Predictably Generate Drug Tolerance and Resistance
Source: mBio. 2022 Nov 8;13(6):e02795-22. doi: 10.1128/mbio.02795-22 (PMC9765434; doi:10.1128/mbio.02795-22)
Supplement: FIG S3 [file mbio.02795-22-s0003.pdf]

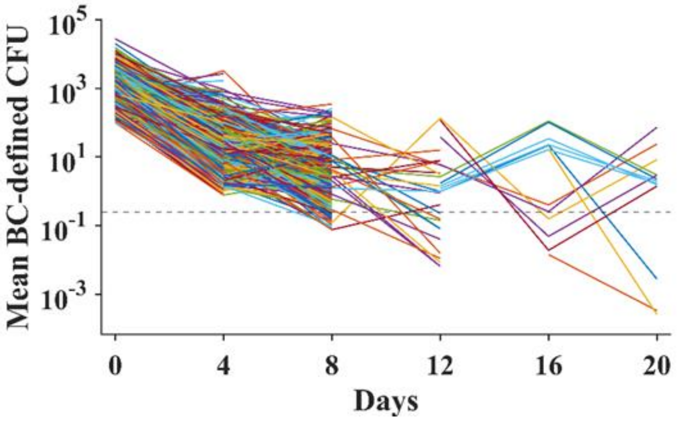

Fig. S3. Barcode level time-kill kinetics of Experiment 3. Individual barcode count kill curves, adjusted for the number of CFU in each assay well, were generated in all replicate culture wells at each time point. Each line represents the trajectory of a unique barcode. All barcode reads above the 10 barcode count per well cut-off were included in the analysis; however, mean barcodes lower than one are reported (below the dotted line) when this is due to averaging barcode numbers across wells. Cultures with rifampicin resistance were excluded from the analysis.
