# Supplementary material for: Origin and Dynamics of Mycobacterium tuberculosis Subpopulations That Predictably Generate Drug Tolerance and Resistance
Source: mBio. 2022 Nov 8;13(6):e02795-22. doi: 10.1128/mbio.02795-22 (PMC9765434; doi:10.1128/mbio.02795-22)
Supplement: FIG S4 [file mbio.02795-22-s0004.pdf]

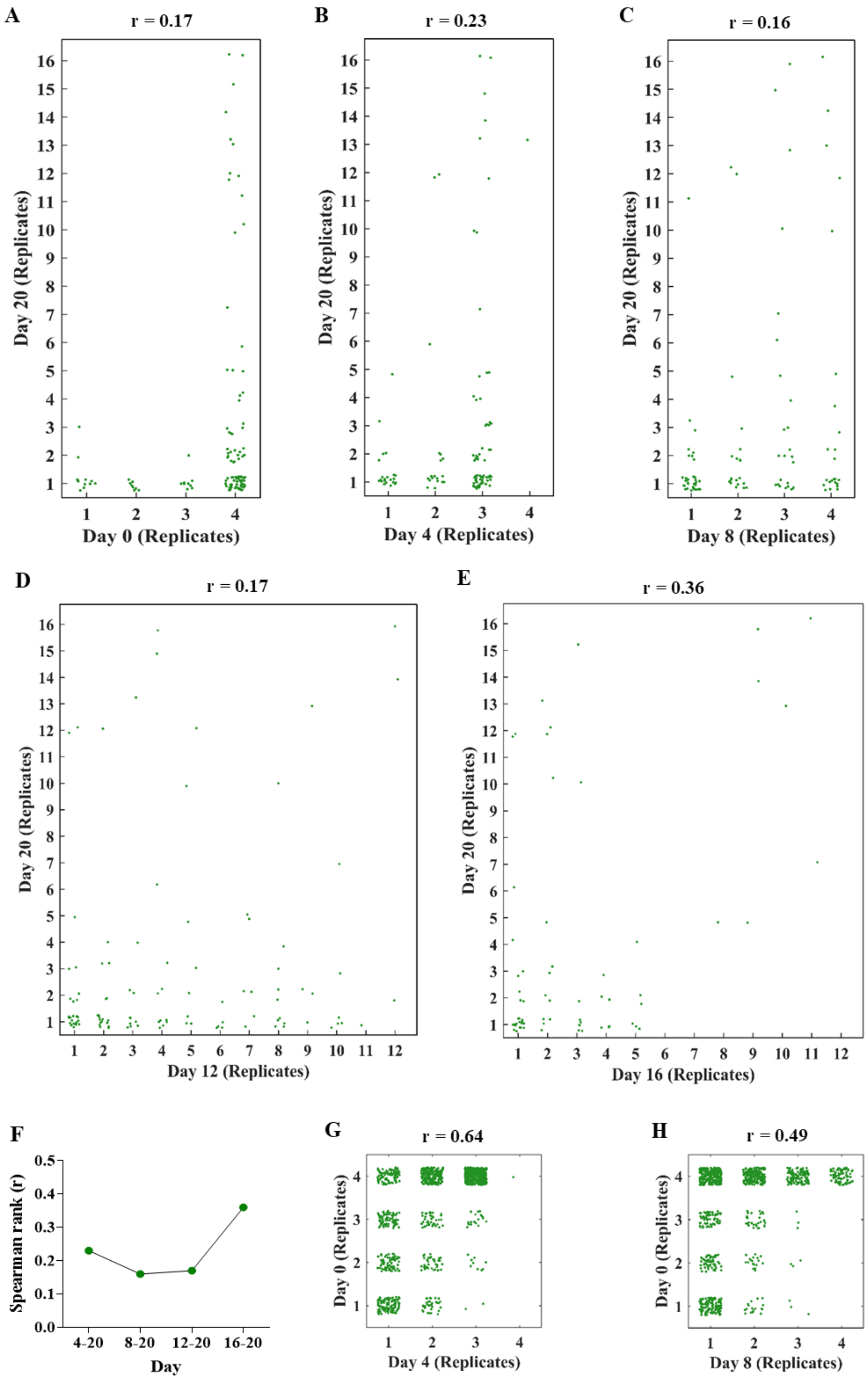

A
r = 0.17
Day 20 (Replicates)
Day 0 (Replicates)
B
r = 0.23
Day 20 (Replicates)
Day 4 (Replicates)
C
r = 0.16
Day 20 (Replicates)
Day 8 (Replicates)
D
r = 0.17
Day 20 (Replicates)
Day 12 (Replicates)
E
r = 0.36
Day 20 (Replicates)
Day 16 (Replicates)
F
Spearman rank (r)
4-20
8-20
12-20
16-20
Day
G
r = 0.64
Day 0 (Replicates)
Day 4 (Replicates)
H
r = 0.49
Day 0 (Replicates)
Day 8 (Replicates)

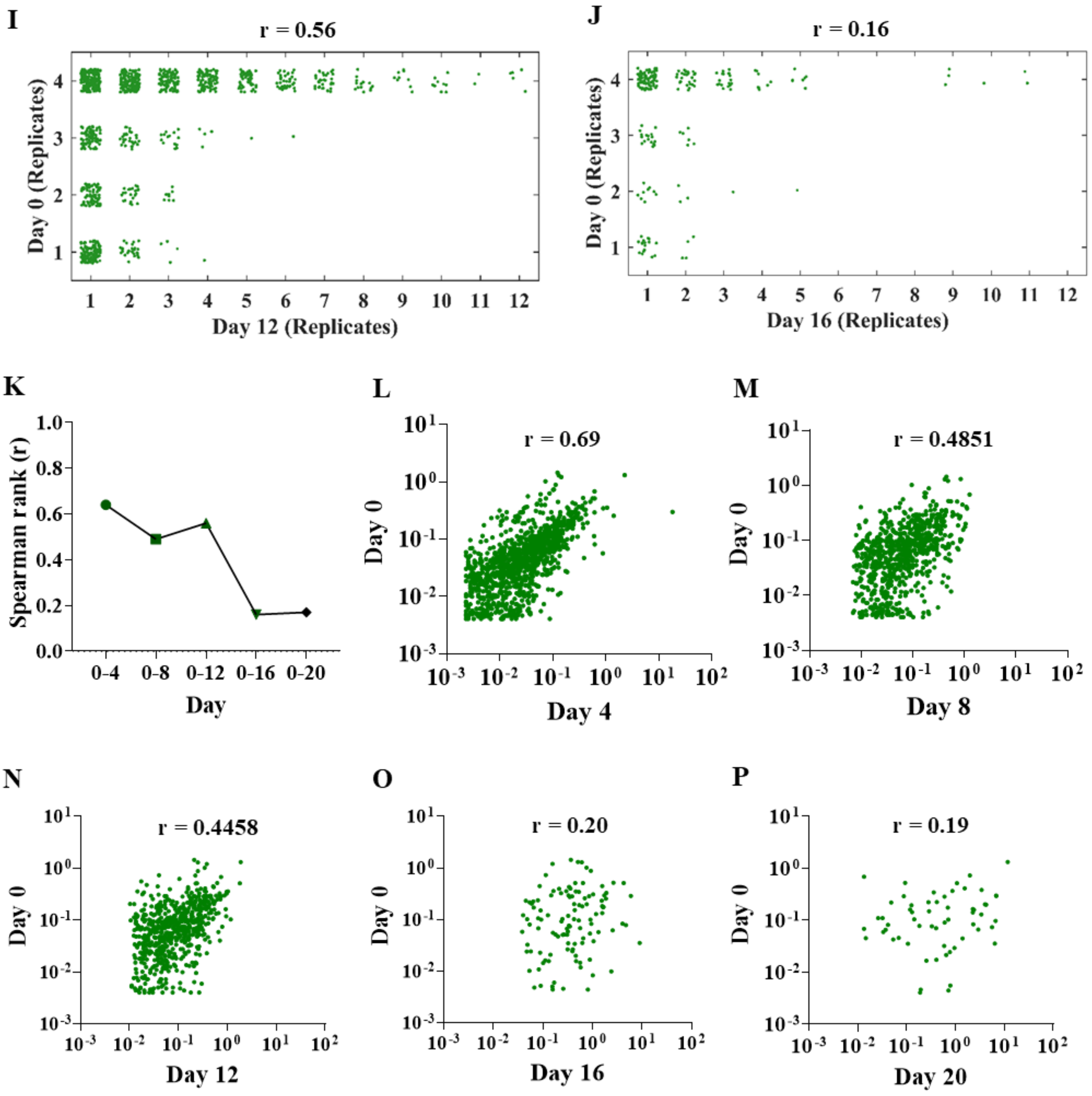

Fig. S4. Enrichment of tolerant forms during extended rifampicin exposure is reflected by an increased correlation between samples at later time points compared to early time points. Experiment 2 results. A-E Individual barcode plots and Spearman rank correlations (r) between the number of wells in which each unique barcode is found at day 20 versus each earlier time point. Only barcodes that were present in at least one well on the two comparison days are shown. F, Summary plot of all Spearman rank correlations from panels A-E. G-J, Individual

barcode plots and Spearman rank correlations between the number of wells in which each unique barcode is found at day 0 versus each later time point. Only barcodes that were present in at least one well on the two comparison days are shown. K, Summary plot of all Spearman rank correlations from panels G-J plus E. L-P Spearman rank correlations of barcode frequency between day 0 versus each later time point. Each unique barcode that was present in at least one well at each time point is shown by a dot and the frequency for each barcode represents a mean frequency of all wells at that time point. Spearmen rank correlations were calculated using GraphPad Prism9.1.
