# Supplementary material for: Origin and Dynamics of Mycobacterium tuberculosis Subpopulations That Predictably Generate Drug Tolerance and Resistance
Source: mBio. 2022 Nov 8;13(6):e02795-22. doi: 10.1128/mbio.02795-22 (PMC9765434; doi:10.1128/mbio.02795-22)
Supplement: FIG S6 [file mbio.02795-22-s0006.pdf]

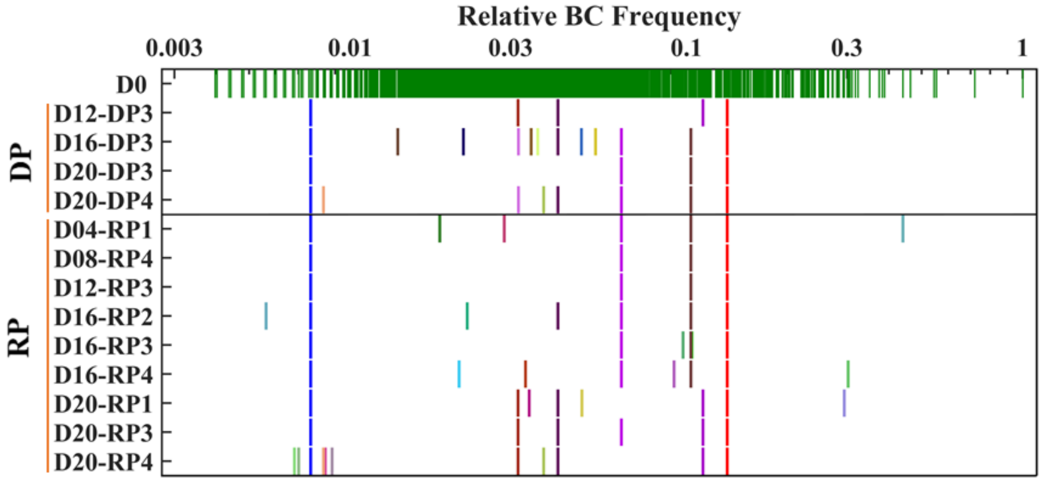

Fig. S6. Diversity and conservation of barcodes detected in drug resistant CFU for Experiment 3. All barcodes detected on day 0 are distributed across the top row of the panel (green lines) according to their mean relative frequency at day 0 (from low to high frequency). All of the barcodes identified in resistant CFU are assigned a specific color and position that is aligned in the figure according to their mean frequency in drug susceptible day 0 wells. Barcodes found in resistant DP or FP cultures are indicated. Color and position denote a unique barcode. When resistance barcodes were detected in independent cultures they are show in the same color and X axis position. The Y axis denotes cultures named with the day of treatment and replicate number.
