# Supplementary material for: Origin and Dynamics of Mycobacterium tuberculosis Subpopulations That Predictably Generate Drug Tolerance and Resistance
Source: mBio. 2022 Nov 8;13(6):e02795-22. doi: 10.1128/mbio.02795-22 (PMC9765434; doi:10.1128/mbio.02795-22)
Supplement: FIG S7 [file mbio.02795-22-s0007.pdf]

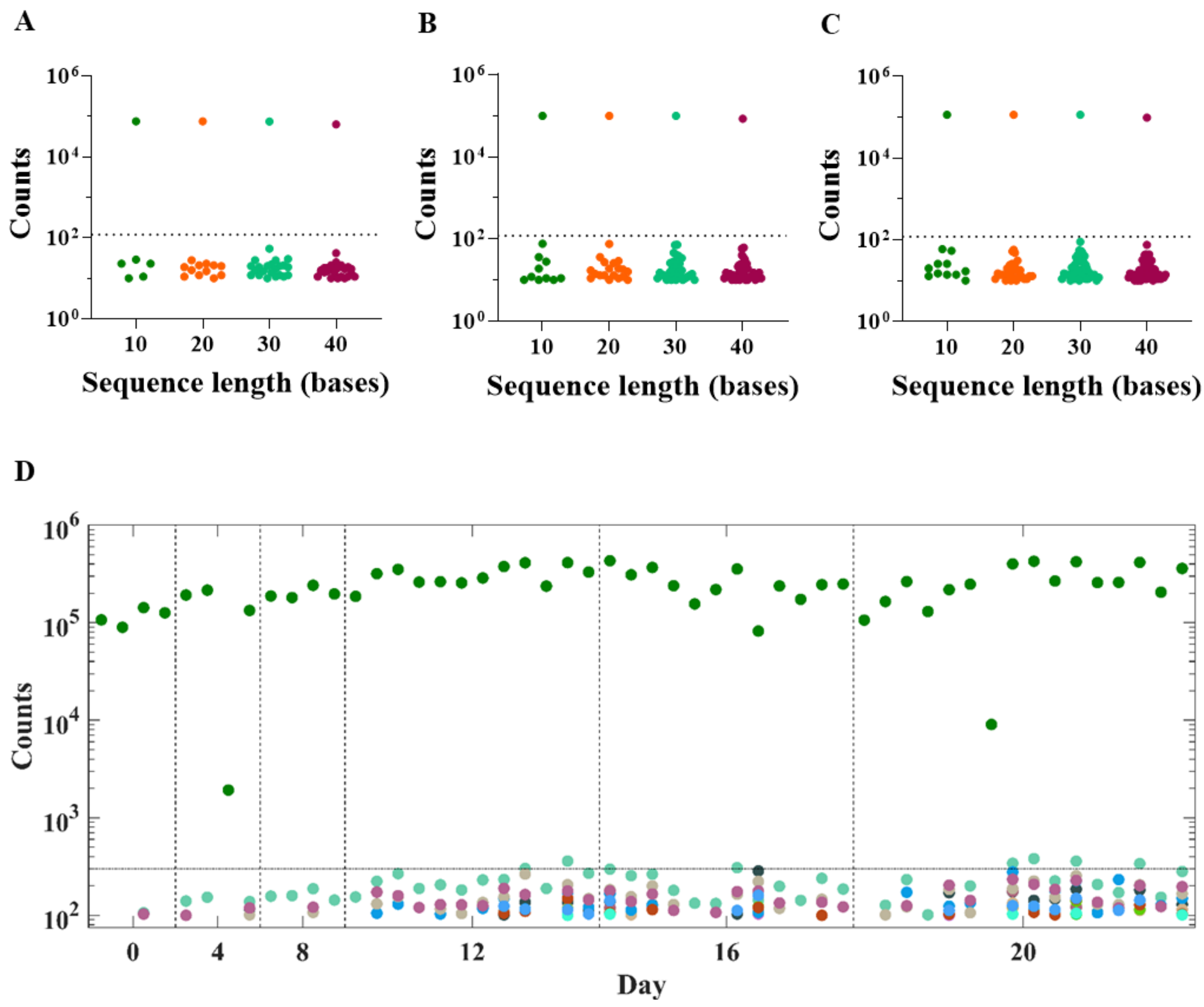

Fig. S7. Estimating deep sequencing fidelity. Non-barcode region (flanking sequence) of barcode deep sequence reads from three cultures before drug exposure (S1, S2, S3) were extracted at various lengths (X axis) to estimate the number of sequence variants and their counts (Y axis) A, B, C. The number of variants increases with sequence length whereas the counts did not show a corresponding increase. D, Dot plot of the 40 base long non-barcode region from the same set of no drug (day 0) and rifampicin treated samples (days 4 – 20) subjected to RRDR sequencing suggested 120 read counts (dotted line) as the correct stringency cut-off limit for detecting specific RRDR unfixed resistance mutations.
