# Supplementary material for: Origin and Dynamics of Mycobacterium tuberculosis Subpopulations That Predictably Generate Drug Tolerance and Resistance
Source: mBio. 2022 Nov 8;13(6):e02795-22. doi: 10.1128/mbio.02795-22 (PMC9765434; doi:10.1128/mbio.02795-22)
Supplement: FIG S8 [file mbio.02795-22-s0008.pdf]

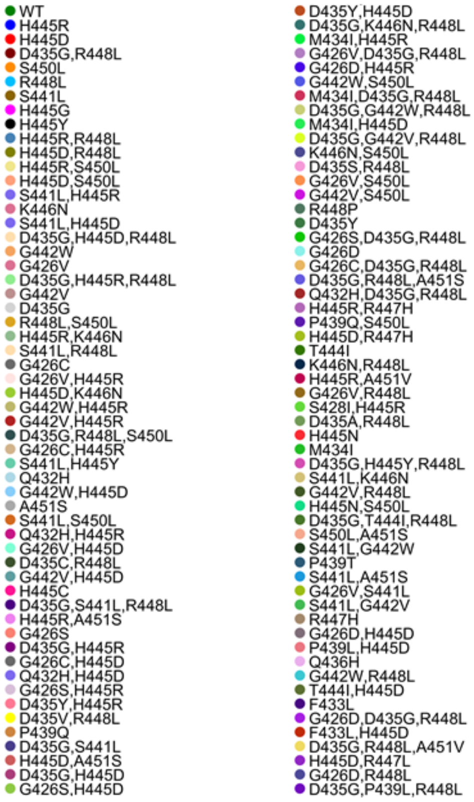

Fig. S8. List of amino acid variants observed by deep sequence analysis of the RRDR locus of the *rpoB* gene from the samples in Experiment 2.
