# Supplementary material for: Origin and Dynamics of Mycobacterium tuberculosis Subpopulations That Predictably Generate Drug Tolerance and Resistance
Source: mBio. 2022 Nov 8;13(6):e02795-22. doi: 10.1128/mbio.02795-22 (PMC9765434; doi:10.1128/mbio.02795-22)
Supplement: TABLE S1 [file mbio.02795-22-s0010.pdf]

RRDR variants associated with Rifampicin resistance[23]	Other RRDR variants with possible Rifampicin resistance
A451V	A451S
D435A	D435C
D435G	D435S
D435V	F433L
D435Y	G426C
G426S	G426V
H445C	G442V
H445D	G442W
H445G	K446N
H445N	P439Q
H445R	P439T
H445Y	Q436H
M434I	R447H
P439L	R447L
Q432H	R448L
S428I	R448P
S441L	
S450L	
T444I	

Table S1. List of clinically relevant RRDR variants associated with rifampicin resistance in *M. tuberculosis* isolates along with the additional variants observed by deep sequence analysis of the RRDR locus of the *rpoB* gene from Experiment 2.
