# Supplementary material for: Origin and Dynamics of Mycobacterium tuberculosis Subpopulations That Predictably Generate Drug Tolerance and Resistance
Source: mBio. 2022 Nov 8;13(6):e02795-22. doi: 10.1128/mbio.02795-22 (PMC9765434; doi:10.1128/mbio.02795-22)
Supplement: FIG S9 [file mbio.02795-22-s0009.pdf]

A

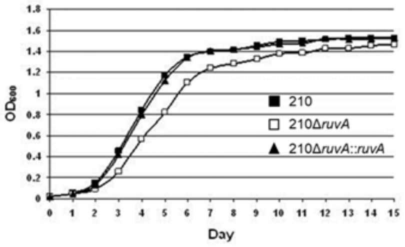

B

	MIC (µg/ml)							MIC (mM)
	INH	RIF	EMB	KAN	CIP	MMC	MMS	H2O2
**210**	0.05	0.05	2	2.5	0.3	20	512	2.5
**210ΔruvA**	0.05	0.05	2	2.5	0.1	8	128	1.25
**210ΔruvA::ruvA**	0.05	0.05	2	2.5	0.3	20	512	2.5

C

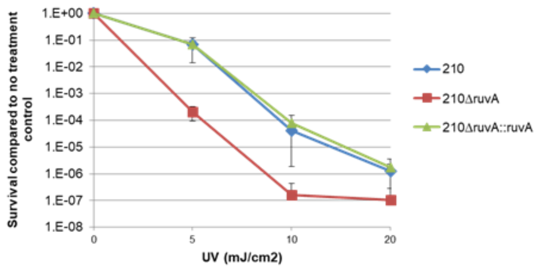

D

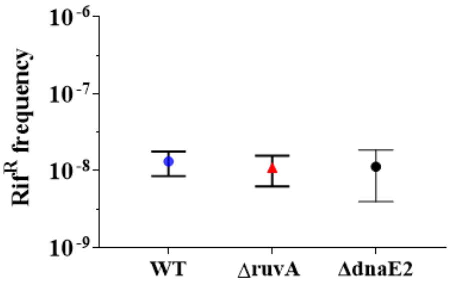

Fig. S9. **A**, Growth curve of wild-type *M. tuberculosis* (210 strain), *ruvA* deletion mutant (210::Δ*ruvA*) and the complemented *ruvA* deletion mutant (210Δ*ruvA*::*ruvA*). Cultures were grown in 7H9 medium supplemented with OADC (10%) and Tween 80 (0.05%), and the OD_{600} was measured every 24 hours. **B**, MIC measurements were performed by 7H10 agar proportion and BACTEC 460TB as previously described. Both methods produced identical values for each strain. (INH: isoniazid; RIF: rifampin; EMB: ethambutol; Kana: kanamycin; CIP: ciprofloxacin; MMC: mitomycin C; MMS: methyl methanesulfonate; H2O2: hydrogen peroxide). **C**, Hypersusceptibility of *ruvA* mutant to UV light. Serial dilutions of *M. tuberculosis* mid-log phase cultures were plated onto 7H10 agar medium and exposed to UV light using the Spectrolinker XL-1000 UV crosslinker apparatus (Spectronics Corporation) in a level 3 biosafety cabinet. The plates were then incubated for 4 weeks and CFU counts were compared to control plates. **D**, Natural rifampicin resistance frequency of wild-type $Mc^2$6230 and the knock-out mutants for *ruvA* and *dnaE2*.
